# Iodine-Supported Hip Implants: Short Term Clinical Results

**DOI:** 10.1155/2015/368124

**Published:** 2015-10-28

**Authors:** Tamon Kabata, Toru Maeda, Yoshitomo Kajino, Kazuhiro Hasegawa, Daisuke Inoue, Takashi Yamamoto, Tomoharu Takagi, Takaaki Ohmori, Hiroyuki Tsuchiya

**Affiliations:** Department of Orthopaedic Surgery, Kanazawa University Graduate School of Medical Science, 13-1 Takara-machi, Kanazawa 920-8641, Japan

## Abstract

We developed a new povidone iodine coating technology for titanium hip implants and performed a clinical trial to assess its usefulness in suppressing postoperative infection. Results indicate that iodine-supported titanium has favorable antibacterial activity, biocompatibility, and no cytotoxicity. Thirty joints in 28 patients were treated using iodine-supported implants. Fourteen joints were revision total hip arthroplasty (THA) after periprosthetic infection, 13 were primary THA for immunosuppressive conditions or pyogenic arthritis, and 3 were conversions from hemiarthroplasty to THA for immunosuppressive conditions. Two examinations were conducted sequentially until final follow-up: white blood cell (WBC) and C-reactive protein (CRP) were measured pre- and postoperatively and thyroid hormone levels in the blood were examined. The mean follow-up period was 33 months (14–78). There were no signs of infection in any patient at the last follow-up. WBC and CRP levels returned to normal within several weeks. No abnormalities of thyroid gland function were detected. Loosening of the implants did not occur in any patient. Excellent bone ingrowth and ongrowth were found around prostheses. No cytotoxicity or adverse effects were detected. These results suggest that iodine-supported THA implants can be highly effective in preventing and treating postoperative infections.

## 1. Introduction

Total hip arthroplasty (THA) is frequently used to treat degenerative hip joint diseases and is widely recognized as one of the most successful orthopedic surgeries. The increase in average life span has resulted in a dramatic increase in the number of THAs, which is estimated to exceed 500,000 per year by the year 2020 [1]. This will also mean an increase in the number of periprosthetic joint infections (PJI). PJI is a common complication after THA. The incidence is approximately 1% after primary cases and about 4% in revision cases, despite strict antiseptic operative procedures which include systemic prophylaxis [2, 3]. Currently, PJI is the second or third most common cause for revision hip arthroplasty [4, 5].

Postoperative PJIs are thought to be the result of bacterial adhesion to the implant surface and subsequent biofilm formation at the implantation site. In order to decrease bacterial infection, it is crucial to inhibit bacterial adhesion to the implant surface since biofilm can be very resistant to immune response and antibiotics. To prevent bacterial colonization and biofilm formation, several antibacterial coatings have been proposed, including vancomycin [6], gentamicin [7], carbonated hydroxyapatite (HA) [8], nitric oxide-releasing xerogel [9], and silver [10]. However, available technologies are far from ready for large-scale application, due to various limitations such as questionable long-term effects on bacterial resistance, osseointegration, regulatory issues, and cost.

We recently developed a new procedure for the anodization of povidone-iodine-containing surfaces that could be directly supported on existing implants [11, 12]. Iodine is a component of thyroid hormones and is the heaviest essential element needed by all living organisms. Our previous studies indicate that iodine-supported titanium has antibacterial activity, biocompatibility, and no cytotoxicity [11].

In this study, we performed a clinical trial of the newly invented technology of povidone iodine-supported THA implants.

## 2. Materials and Methods

A consecutive single-center series of 30 joints in 28 patients treated with iodine-supported hip implants was reviewed. The study group consisted of 13 men and 15 women with a mean age of 56 years (range, 17–81 years). Follow-up after surgical intervention averaged 33 months (range, 14–78 months). Fourteen joints were revision THA after PJI, and 13 joints were primary THA for compromised immune system conditions (severe diabetes mellitus, high dose glucocorticoid administration, under chemotherapy, inactive infections in other organs, and so on) or pyogenic arthritis. Three joints were conversions from hemiarthroplasty (bipolar in two, monopolar in one) to THA for immunosuppressive conditions. In the revision THAs after PJI, only one case underwent single-stage revision, while the other 13 needed two-stage revisions using antibiotic-loaded acrylic cement spacers (ALAC). In primary THA for pyogenic arthritis, 4 cases needed two-stage surgery using ALAC, while one case underwent single-stage implantation. Postoperatively, all patients received antibiotics intravenously tailored to the sensitivities of intraoperative cultures for at least 3–7 days, followed by oral administration until inflammatory markers (CRP, ESR) return to stable limits.

**Figure 1:**
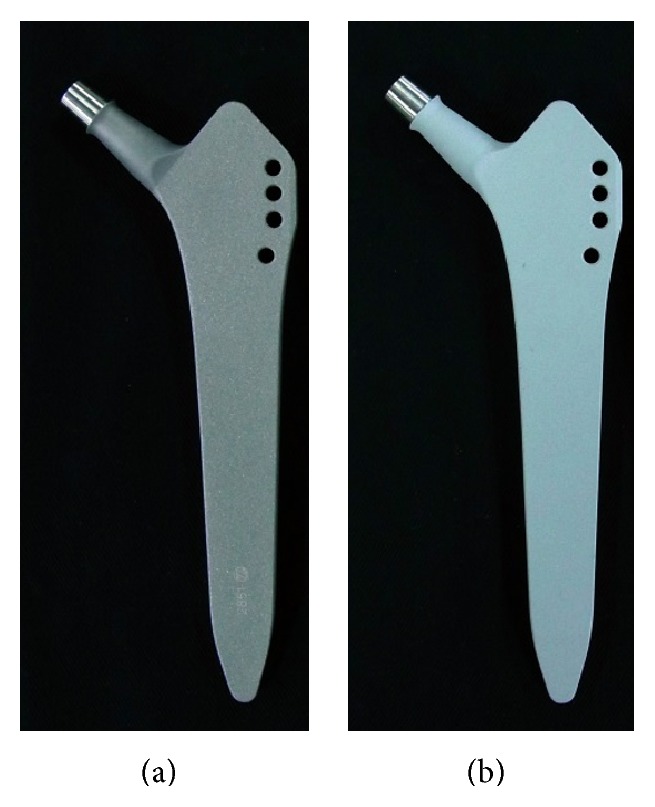
An uncoated hip prosthesis (a) and an iodine-supported hip prosthesis (b).

**Figure 2:**
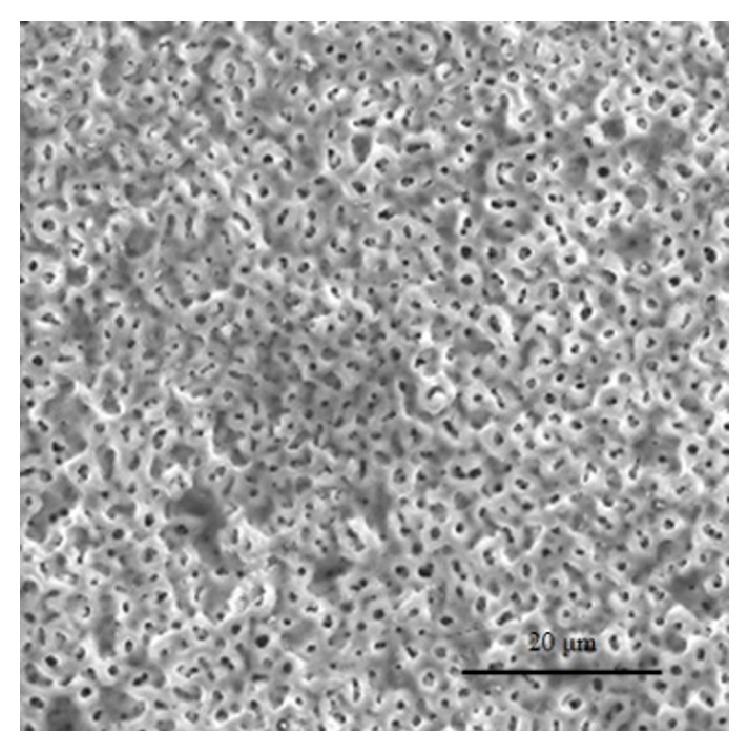
Electron micrograph of the oxide layer: more than 50,000 pores/mm^2^.

In all cases in this series we used titanium hip implants with iodine-containing surfaces (Figure 1). Two weeks before surgery, all implants were selected according to the preoperative plan using a 3D templating system (ZedHip, Lexi Co., Tokyo, Japan), and iodine-containing surface treatments were applied by the Chiba Institute of Technology (Narashino, Japan) using the technique described by Hashimoto et al. [13]. The thickness of the anodic oxide film was 5–10 *μ*m with >50,000 pores/mm^2^, with the capacity to support 10–12 *μ*g/cm^2^ of iodine (Figure 2) [11, 12]. Twenty-six acetabular sockets (Trilogy, Zimmer, Warsaw, USA; Converge, Zimmer, Warsaw, USA; Tritanium, Stryker, Mahwah, USA), 4 acetabular reinforcement cages (KT-plate, Kyocera, Osaka, Japan; Contour, Smith & Nephew, Memphis, USA), and 26 femoral stems (Allo-classic, Zimmer, Warsaw, USA; CLS, Zimmer, Warsaw, USA; Mayo conservative hip, Zimmer, Warsaw, USA; S-ROM-A, DePuy-Synthes Warsaw, USA) were iodine-supported and implanted.

Perioperatively, white blood cell (WBC) counts and C-reactive protein (CRP) levels were analyzed. To assess the influence of iodine from the implant, thyroid hormone levels in the blood, including thyroid-stimulating hormone (TSH), free triiodothyronine (FT3), and free thyroxine (FT4), were examined. Postoperative radiological evaluations were performed regularly, during which time loosening and failure of the implants were checked.

This study was approved by the ethics committee of our university. Written informed consent was obtained from all 28 patients.

## 3. Results 

**Figure 3:**
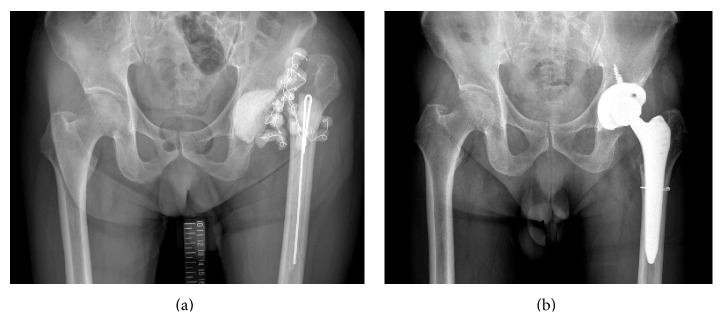
Case of a 65-year-old man. PJI with methicillin-resistant coagulase-negative staphylococcus. Previous treatments including single-stage revision, implant removal, several irrigation, debridement, and ALAC treatments were performed by former surgeon, but suppression of PJI could not be obtained (a). Before iodine-supported prosthesis implantation, inflammation was still active, but the infection was cured after thorough debridement and implantation of iodine-supported hip prosthesis. CRP was 0.1 mg/dL and WBC was 4,400/ll 12 months later (b).

In the series of revision THAs after PJI, isolated microorganisms included methicillin-resistant coagulase-negative* Staphylococci* (MRCNS) in 6 patients, methicillin-resistant* Staphylococcus aureus* (MRSA) in 1 patient, and unknown organisms in 6. One case underwent single-stage revision because of the patient's advanced age and general status. Thirteen cases underwent a two-stage ALAC treatment before implantation, which was effective in 10 cases but could not control PJI in the remaining 3 (Figure 3). Regardless of the preoperative treatment for PJI, complete suppression of PJI was obtained at the final evaluation in all cases except one pelvic tumor reconstruction case, which showed a slight elevation of CRP levels at 24 months after temporary suppression was obtained.

**Figure 4:**
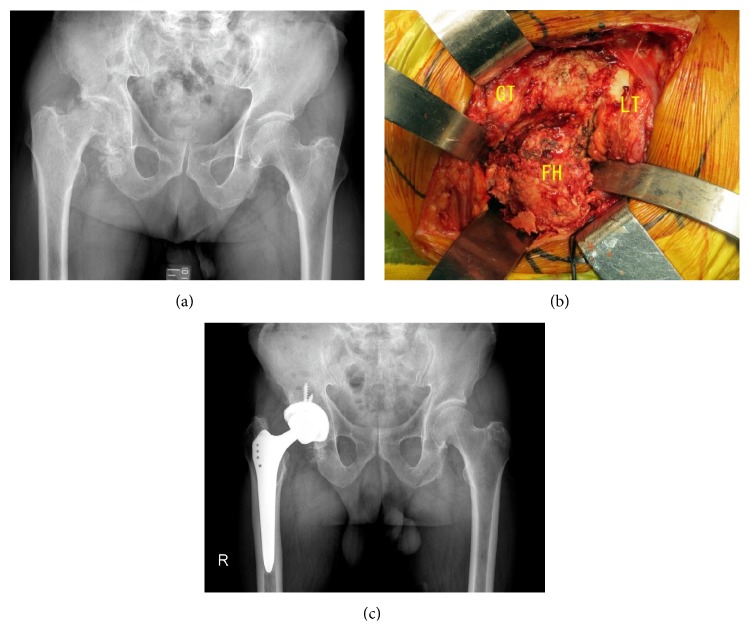
Case of an 82-year-old man. Pyogenic arthritis of the right hip joint with methicillin-sensitive* Staphylococcus aureus* (MSSA). Irrigation and antibiotics treatment in former hospital could not suppress infection, which resulted in femoral head destruction (a). Right hip joint was filled with infected scar tissue and debris of the destructed femoral head. Single-stage implantation was performed after thorough debridement (b). GT: greater trochanter, LT: lesser trochanter, and FH: femoral head. One year after surgery, his hip function was almost normal, and iodine-supported hip prosthesis was well fixed. CRP was 0.2 mg/dL and WBC was 3,810/ll 12 months later (c).

In the series of primary THAs for compromised immune conditions, PJI was prevented in all 11 cases. In the series of pyogenic arthritis, isolated microorganisms included methicillin-sensitive* Staphylococcus aureus* (MSSA) in 3 patients,* Pseudomonas aeruginosa* in 1, and* Streptococcus agalactiae* in 1. Although 1 case with* Pseudomonas aeruginosa* needed additional wound irrigation because of continuous wound discharge, all 5 cases successfully underwent primary THA without recurrence of infectious arthritis (Figure 4).

As for postoperative complications, three cases had postoperative dislocation, all of which were conservatively treated. None of the implants loosened during the follow-up period. Radiography revealed excellent bone ingrowth and ongrowth around the prostheses except for two acetabular sockets which showed slight radiolucent lines.

**Figure 5:**
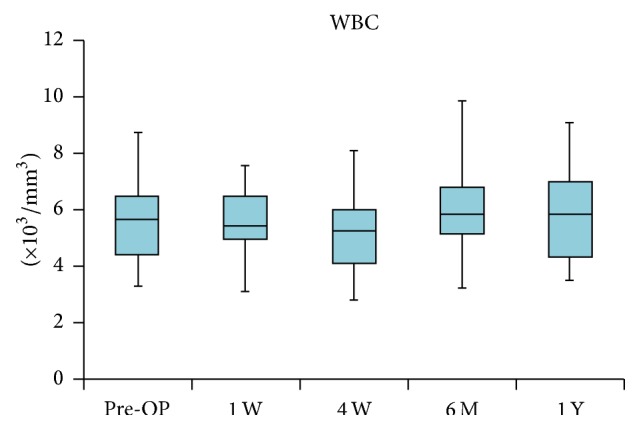
Changes in white blood cell (WBC) counts (/mm^3^). WBC counts tended to be high several days after the surgery but returned to normal by 1 or 2 weeks.

**Figure 6:**
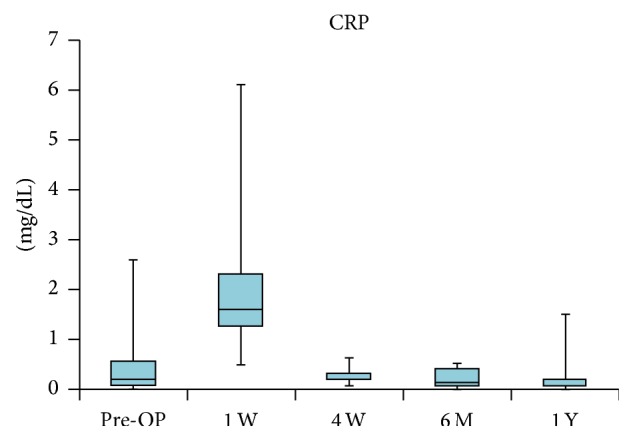
Changes in C-reactive protein (CRP; mg/dL). CRP level returned to normal level within 4 weeks after implantation.

In the antibacterial treatment cases (14 cases of PJI and 5 cases of pyogenic arthritis), the preoperative median WBC counts and median CRP levels were 5,670/mm^3^ (range, 3,630–8,690/mm^3^) and 0.2 mg/dL (range, 0.0–2.6, reference value 0.3), respectively. Although the WBC counts tended to be high one week after surgery, they returned to normal by the final evaluation (median 5,860/mm^3^, range 3,490–9,040/mm^3^; median 0.2 mg/dL, range 0.0–1.5) (Figures 5 and 6).

**Figure 7:**
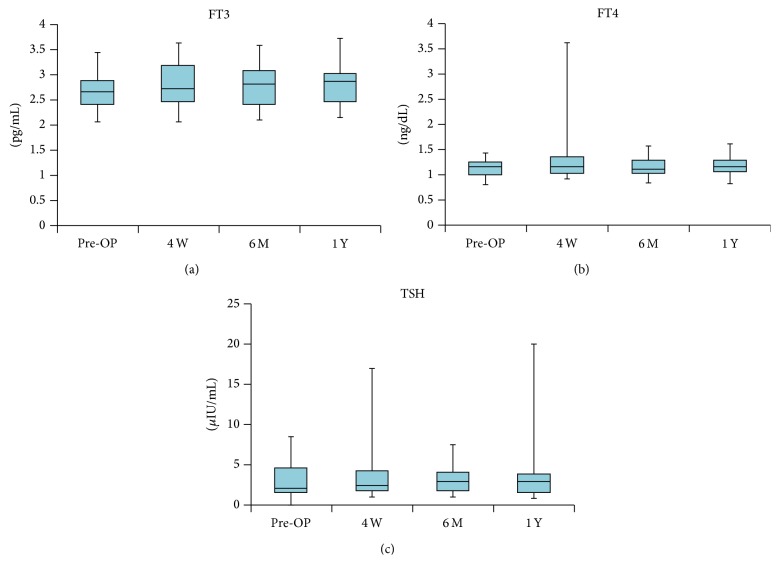
The free triiodothyronine (FT3; pg/mL), free thyroxine (FT4; ng/dL), and thyroid-stimulating hormone (TSH; *μ*IU/mL) levels were within the normal ranges during the study period.

In all cases, the preoperative FT3 (pg/mL), FT4 (ng/dL), and TSH levels (*μ*IU/mL) were almost normal ranges (median 2.68 pg/mL, range 2.07–3.43; 1.14 ng/dL, range 0.8–1.42; 1.98 *μ*IU/mL, range 0.3–8.54, resp.). One year postoperatively, the levels were still within the normal ranges (median 2.87 pg/mL, range 2.17–3.73; 1.14 ng/dL, range 0.81–1.59; 2.9 *μ*IU/mL, range 0.86–19.9, resp.) (Figure 7). There was no case of thyroid gland malfunction with the use of iodine-supported implants.

## 4. Discussion

PJI represents one of the most severe complications in joint arthroplasty. Once severe PJI occurs, the cure rate by conservative treatment is low, and some kind of surgical intervention, such as removal of the implants, is usually required. However, an extremely effective prophylaxis or effective treatment for PJI has not yet been established. Systemic administration of antibiotics for bacterial infection is the most common therapy but is not effective for the resistant bacteria. Also, the local soft tissue surrounding implant cannot supply an effective amount of antibiotics for the bacteria present in the implant surface because of the lack of blood flow. Therefore, it would be meaningful from the viewpoint of PJI prevention and treatment for the surface of the implant to possess local antibacterial activity. Various strategies to provide implants with an antibacterial coating have been proposed recently [6–12].

In this study, we used iodine-supported titanium hip implants. Clinically, the iodine coating has several advantages. First, the antibacterial spectrum of iodine is very broad, acting not only on general bacteria but also on certain viruses, tubercle bacilli, and fungi [12]. Second, iodine does not cause drug resistance as it might be induced by the administration of antibiotics [12]; it has been noted that PJI due to antibiotic resistant bacteria is increasing [14], and antibiotic coating implants, such as gentamicin, may not sufficiently prevent PJI. Third, iodine is a trace metal and an essential component of the thyroid hormone. Iodine released from the implant is biologically safe because it can be excreted by the kidneys. In this study, TSH, FT3, and FT4 levels were in the normal range during the study period, postoperative WBC and CRP levels had returned to normal by the end of the study period, and no abnormalities of thyroid gland function were detected (Figure 7). Fourth, duration of the antibiotic effect is relatively long. The biological half-life of an antibiotics coating or silver coating is said to be only several hours or days [15, 16]. On the other hand, recent data showed that the amount of iodine on the titanium pin in patients with external fixation was maintained, with approximately 40% remaining after 1 year [17]. Fifth, iodine-supported implants have excellent osteoconduction and good biocompatibility [11]; bone conduction is reportedly not possible on certain materials such as silver [10]. In this study, radiography showed excellent bone ingrowth and ongrowth. There were no signs of bone absorption or inhibition of bone formation. We think that the osteoconductive effects were reinforced according to the porous structure of the oxide layer. The characteristics of these iodine prosthesis coatings are extremely advantageous for PJI treatment. Although the number of cases was small, the results of this clinical trial suggest that iodine-supported titanium hip implants can be very effective in the prevention and treatment of PJIs after hip arthroplasty.

We think that the introduction of iodine coating for hip implants could result in a paradigm shift in current treatment strategies for PJI of the hip. Today two-stage revision arthroplasty is regarded as the gold standard treatment method for severe PJI [18]. Nevertheless, this procedure is costly and time-consuming and may affect the patient's mental condition. In contrast to two-stage revision, single-stage revision offers a shorter hospital stay, the avoidance of complications associated with a second operation, improved postoperative function and pain, and lower cost. However, this procedure is usually applied for highly selected patients. Using a reimplanted hip prosthesis that is iodine-supported could broaden the single-stage revision options. In this series, we successfully performed a single-stage revision for a PJI case in an elderly patient and a single-stage implantation for pyogenic arthritis. Our recent report demonstrated the efficacy of iodine-supported titanium implants in the management of active pyogenic vertebral osteomyelitis [19].

This case series does not have a sufficient number of cases or a long enough follow-up period. However, no infection recurred during the follow-up period in the series of revision THAs after PJI, and in the series of primary THAs for patients with compromised immune conditions, PJI was prevented in all cases. For comparison, a meta-analysis of 36 articles has reported that the infection recurrence rate in two-stage revision for PJI is 10.3%, while the infection rate for single-stage revision is 13.1% [20]. Also, it is well-known that joint replacement surgery for compromised immune conditions has a higher rate of PJI than for healthy patients. The reality is that, in these cases we cannot completely prevent PJI. However, the iodine coated implant is thought to have significant potential for reducing those rates of infection.

On the other hand, it must be recognized that there are several disadvantages to iodine-supported implants. The iodine coating procedure is costly and time-consuming (about 10 days for preparation). It cannot be applied to implants made from CoCr or ceramics, so the joint surface usually remains noncoated material. The implant size must be selected before surgery, which requires perfect preoperative planning. We routinely use a 3D templating system in the preoperative planning to select the appropriate implant design and size. The coating makes polished surfaces rough, which may influence the original implant design concepts of each implant. We prefer to select an implant with a fully rough surface, like a Zweymuller type stem with a grit-blasted surface.

The present study has several limitations, including the uncontrolled retrospective design, small sample size, and diversity of patient background. Therefore, a prospective randomized clinical trial on a large scale is necessary to demonstrate the statistical significance of the infection rate. Although the small patient size limits the significance of the present study, the positive effect of iodine-supported implants in the prevention or treatment of PJI after THA is encouraging and merits further investigation.

## 5. Conclusion

The results of this clinical trial suggest that iodine-supported THA implants can be very effective and show great promise in the prevention and treatment of PJIs.
